# Prophylaxis protects infants with congenital heart disease from severe forms of RSV infection: an Italian observational retrospective study: Palivizumab prophylaxis in children with congenital heart disease

**DOI:** 10.1186/s13052-022-01399-z

**Published:** 2023-01-11

**Authors:** Chiara Ratti, Anna della Greca, Deborah Bertoncelli, Monica Rubini, Bertrand Tchana

**Affiliations:** 1 Pediatric Cardiology Unit, General and University Hospital of Parma, Parma, Italy; 2 General and Emergency Pediatric Unit, General and University Hospital of Parma, Parma, Italy

**Keywords:** Palivizumab, Congenital heart disease, RSV infection, RSV prophylaxis, Respiratory syncytial virus, Hospitalization, Infants with CHD

## Abstract

**Background:**

In children with congenital heart disease (CHD) respiratory syncytial virus (RSV) infection may have a severe course, with increased risk of morbidity and mortality, requiring hospital admission and intensive care. The aim of the present study was to evaluate the effect of prophylaxis with palivizumab in preventing RSV-associated hospitalization in infants with CHD.

**Methods:**

We carried out an observational, retrospective study in a paediatric cardiology division at a secondary-care centre in Italy, extracting from the database children with CHD who, from November 2004 to March 2022, matched the criteria for palivizumab prophylaxis, to evaluate the hospitalization rate in CHD patients with and without palivizumab prophylaxis and their RSV-related hospitalization characteristics compared with a group of children without CHD and no other underlying clinical conditions (control group, CG), hospitalized for RSV infection.

**Results:**

One hundred twenty-eight children with CHD were enrolled in the study, mainly (71.9%) with increased pulmonary flow, and received palivizumab prophylaxis. Twenty-seven received hospital care for bronchiolitis. Almost all CHD patients hospitalized for bronchiolitis (26 out of 27) received partial prophylaxis (≤ 3 doses). CHD patients with bronchiolitis stay longer in the hospital than control (14.4 ± 21.7 days vs 6.2 ± 2.3 days) some of which require intensive care (*n* = 4).

**Conclusions:**

Our study provides evidence of the efficacy of palivizumab in protecting patients with hemodynamically significant CHD under the age of 2 years from RSV disease and its life-threatening complications. Reducing hospitalisation rate, morbidity, and mortality in this category of patients, passive immune prophylaxis with palivizumab may impact healthcare resource availability and utilisation.

## Background

Congenital heart disease (CHD) is a leading cause of infant mortality related to birth defects, with an approximate incidence of 8 per 1,000 live births [1, 2]. Delayed CHD diagnosis and treatment may cause chronic disability and morbidity, dramatically reducing life expectancy of patients. In the last decades remarkable progress in diagnostic tools and surgical technics significantly improved survival of children with CHD, allowing them to reach adulthood [3–5]. Nowadays the majority of newborns with complex CHD survive, who turn one year old reach 16 years of age [6].

It has been speculated that children with CHD may present altered cellular and humoral effectors of innate immunity and have detectable circulating markers of chronic inflammation [7], which may have important clinical implications in the management of CHD and comorbidities. Children with CHD might be therefore more susceptible to common infections [8].

Respiratory syncytial virus (RSV) is the most common cause of low respiratory tract infections in children up to the age of 2 years [9]. RSV infection causes a variety of disease manifestations, such as bronchiolitis and pneumonia, with clinical presentation ranging from mild to severe. In most cases, RSV-related disease heals spontaneously; however, some newborns and infant may require hospitalization, experience severe morbidity and early mortality. Because of their clinical conditions, infants with CHD are at high-risk for RSV infection and related complications, thus at risk of RSV induced hospitalisation; several studies have targeted hospitalization rate due to RSV-infection in CHD children, reporting high rates, however difficult to compare because of differences in study populations, methods used and inclusion criteria [10]. In addition to hospitalisation, serious acute forms may need strict follow-up to monitor long-term morbidities [11].

To date, there is no specific treatment for RSV infection, and disease management is based on control of the symptoms, although the severe forms require supportive measures, such as oxygen supplementation or other type of respiratory support [12]. Therefore, the best strategy to limit the spread of RSV infection and protect patients at risk of severe complications is the adoption of preventive strategies of immunization.

RSV epidemics show different seasonal cycles, with a peak during winter months in temperate regions, such as Italy [13]. So far, the only agent available for seasonal prophylaxis of RSV infections is palivizumab, a mAb that recognizes an antigenic site of the F glycoprotein of RSV [14]. In Italy, prophylaxis with palivizumab is recommended, as indicated by regulatory authority (Regione Emilia-Romagna) and scientific societies (Italian Society of Neonatology, Italian Society of Pediatric Cardiology and Congenital heart Diseases) in children under the age of 2 years with hemodynamically significant CHD (HS-CHD), with congestive heart failure that requires medical therapy, cyanosis with systemic saturation less than 90%, or pulmonary hypertension, and in children post cardiac transplantation; prophylaxis in the 2nd year of life is recommended when there is still a need of medical therapy on an ongoing basis [15, 16].

We report on a retrospective analysis of a group of patients with HS-CHD from a single center who received palivizumab prophylaxis, to evaluate its effect in preventing RSV-associated hospitalization.

## Methods

### Study design and patients

This was a single center, observational, retrospective study conducted in the Paediatric Cardiology Division in a secondary-care Hospital in Italy. The study was approved by the Local Health Authority, and the Site’s Ethics Committee approved the request to use each identified patient. Specific written informed consent was obtained from each patient or their parental guardian prior to treatment start.

We searched our database for patients with CHD who underwent prophylaxis with palivizumab to assess it effect in preventing RSV-induce hospitalisation; we also evaluate their hospitalization characteristics and compare them with a group of healthy children (CG) of same age, without CHD or any other clinical condition, hospitalized for bronchiolitis during the same period. CHD patients were divided in groups and subgroups according to the type of congenital heart defect, for statistical studies:

A Congenital heart defects with increased pulmonary flow:
  Simple (AS): large atrial septal defect, ventricular septal defect, large arterial duct
  Multiple (AM): association of at least two simple lesions
  Complex (AC): anomalous pulmonary venous return, atrioventricular canal, aortopulmonary window, truncus arteriosus
B Congenital heart defects with pulmonary hypo-afflux:
  Complex (BC): tetralogy of Fallot, tricuspid atresia, pulmonary atresia, Ebstein anomaly, unilateral absence of a pulmonary artery, anomalous systemic venous return
C Complex congenital heart defects (CC):
  interrupted aortic arch
  transposition of the Great Arteries with ventricular septal defect
  combination of complex lesions:
    Double outlet right ventricle plus interrupted aortic arch
    Double outlet right ventricle plus anomalous pulmonary venous return plus Coarctation of the aorta
    Major aortopulmonary collateral arteries plus ventricular septal defect
  univentricular heart
  primary pulmonary hypertension
D Myocardial disease (DM)

### Assessments and data collection

Patients’ data were collected from the database of the Paediatric Cardiology Unit of Parma General and University Hospital from November 2004 to March 2022. A post-hoc analysis was conducted to determine any possible association between palivizumab prophylaxis of CHD patients and hospitalization. Patients with bronchiolitis were hospitalized according to their need for oxygen therapy (O_2_ saturation, respiratory, and heart rate) and parenteral nutrition. Hospitalization for bronchiolitis was assessed in CHD and CG children.

### Statistical analysis

Continuous variables were given as means with standard deviations (SD) and categorical variables as the number of subjects and percentage values.

Demographic-clinical baseline differences in patients with CHD and control group were tested using Pearson's Chi-square Test (Fisher's exact where appropriate).

Univariate penalized logistic and negative binomial regression models were performed to detect the effect of the demographic-clinical characteristics on hospitalization and high flows outcome, respectively.

Those covariates with a p-value < 0.10 were then selected for the multivariate analysis, where the hospitalization and high flows were the dependent variables. Multivariate analysis was performed using again the penalised logistic and negative binomial regression model for dichotomic and count outcomes, respectively. The model selection was done using the Akaike information criterion and the Likelihood Ratio test was used as the test of statistical significance. The estimated p-values were adjusted for multiple comparisons by the Holm correction method. A *p*-value < 0.05 was considered as significant and data were acquired and analysed in R v4.2.0 software environment [17].

## Results

### Characteristics of the patient population

The study population included 128 infants with HS-CHD, the majority of which (72%) had congenital heart defects with increased pulmonary flow, while CHD with pulmonary, complex CHD and myocardial disease were less represented (Table 1). In about 20% of CHD patients, heart defect was part of a syndrome (Down syndrome; Noonan syndrome; Goldenhar syndrome; DiGeorge syndrome or other genetic disorders). CG consists of 50 children randomly selected who were hospitalized for bronchiolitis.

**Table 1:** Characteristics of the study participants

**Characteristic**	**Descriptive statistics**	
	*CHD patients (n* = *128)*	
**Prophylaxis**	100%	
**Presence of a syndrome**	25 (19.53%)	
**CHD diagnosis**		
*AS*	54 (42.19%)	
*AM*	17 (13.28%)	
*AC*	21 (16.41%)	
*BC*	10 (7.81%)	
*CC*	21 (16.41%)	
*DM*	3 (2.34%)	
*NA*	2 (1.56%)	
**Hospitalization**	27 (21.09%)	

The table presents clinical characteristics of the 128 CHD patients

The results are expressed as the number of subjects with percentage

*NA* Not analyzed

All HS-CHD patients received RSV prophylaxis, of which 26 received only partial prophylaxis (≤ 3 doses) because born at the beginning of the epidemic period, diagnosed during the epidemic period or during the hospitalization for bronchiolitis. Patients who underwent hospitalisation were assessed with a Respiratory Severity Score (RSS), Silverman score for newborns and babies under 3 months of age [18] and a clinical score for babies older than 3 months [19], score that picture, on a scale from 1 to 10, the patients’ condition on admission to the hospital, and useful tool for rapid disease severity assessment in other to decide the correct hospitalization setting (up to intensive care unit) and adequate treatment management as far as the clinical picture may vary and worsen abruptly. The higher the score is the worse are the patient’s conditions. Twenty-seven HS-CHD patients required hospitalisation for bronchiolitis, they showed a higher RSS score compared with CG children (3.2 ± 0.9 and 2.3 ± 0.9 respectively); however, the difference was not statistically significative (*p*-value = 0.1211); 28.6% of CHD patients analysed for access to hospital were admitted in intensive care unit against 10% in the CG group.

### Impact of palivizumab prophylaxis on hospitalization

Twenty-six of the 27 CHD patients hospitalized for bronchiolitis did not receive a complete cycle of prophylaxis (≤ 3 doses). Regarding the length of hospitalization, the univariate negative binomial regression analysis (Table 2) showed a significant association between CHD diagnosis, RSS, PICU and the length of hospitalisation. The subsequent multivariate analysis confirmed a significant association between CHD diagnosis, PICU and the length of hospitalisation (*p*-values: < 0.0001 and 0.0036, respectively) (Table 3). CHD patients who completed a cycle of prophylaxis are less likely to be hospitalized for bronchiolitis (0,99% vs 96,30% *p*-value < 0.0001); CHD patients with an incomplete prophylaxis, besides being more likely to get hospitalized, in case of hospitalization have a longer stay than CG patients (Table 4) (14.4 ± 21.7 days vs 6.2 ± 2.3 days; *p*-value˂ 0.0001).

**Table 2:** Univariate analysis on days of hospitalization (*n* = 57)

**Characteristic**	**Hospitalization days** 8.53 (± 12.15)	**Ratio (95%CI)**	***p*-value**
**CHD diagnosis**			0.0005
*No*	6.16 (± 2.29)	1	
*Increased pulmonary flow (AS* + *AM* + *AC)*	11.71 (± 7.27)	1.9 (1.41: 2.55)	
**RSS**	rho = 0.35	1.2 (1.06: 1.35)	0.0100
**PICU**			0.0001
*No*	6.18 (2.94)	1	
*Yes*	17 (7.07)	2.75 (1.77: 4.20)	
*Neonatology*	9.4 (2.7)	1.52 (1.07: 2.13)	

The results are expressed as mean with standard deviation

rho = Pearson’s correlation coefficients; Ratio (95%CI): exponentiation of the slope/regression coefficient with 95% Confidence Interval; *p*-value: Likelihood Ratio test *p*-value

**Table 3:** Multivariate analysis on the hospitalization days (*n* = 63)

	**Ratio (95%CI)**	***p*-value**
**PICU**		< 0.0001
*No*	1	
*Yes*	1.77 (1.10:2.84)	
*Neonatology*	1.62 (1.16: 2.23)	
**CHD diagnosis**		0.0036
*No*	1	
*Increased pulmonary flow (AS* + *AM* + *AC)*	1.66 (1.19:2.27)	

Ratio (95%CI): exponentiation of the slope/regression coefficient with 95% Confidence Interval; *p*-value: Likelihood Ratio test *p*-value; *PICU* Pediatric Intensive Care Unit

**Table 4:** Analysis on the hospitalization

**Outcome**	**Hospitalized patients** *(n* = *77)*		***p*-value**
	*Control group (n* = *50)*	*CHD patients (n* = *27)*	
**Hospitalization** *(days)*	6.16 (± 2.29)	14.45 (± 21.7)	< 0.0001^a^
**PICU**			0.0010^b^
*No*	45 (90%)	10 (71.43%)	
*Yes*	0 (0%)	4 (28.57%)	
*Neonatology*	5 (10%)	0 (0%)	
**RSS**	2.34 (± 0.92)	3.18 (± 0.87)	0.1211^a^
**Presence of a syndrome**	0 (0%)	8 (29.63%)	
		***Coexisting syndrome among CHD hospitalized patients**** (n* = *27)*	
**Hospitalization** *(days)*	-	*Absence* 15 (± 2.88) *Presence* 13.17 (± 6.77)	0.7662^c^

The table presents the univariate analysis on the hospitalization (control group *n* = 50; CHD patients group *n* = 128)

The results are expressed as the number of subjects with percentage

*RSS* Respiratory Severity Score. This score ranges from 0 to 12 and higher scores imply more severe respiratory disease, *PICU* Pediatric Intensive Care Unit

^a^Control group vs CHD hospitalized patients

^b^PICU Yes/No in Control group vs CHD hospitalized patients

^c^CHD hospitalized with syndrome vs CHD hospitalized without syndrome

The univariate penalized logistic regression analysis demonstrated no significant association between high flow oxygen therapy and any of the demographic-clinical characteristics.

## Discussion

**Fig. 1:**
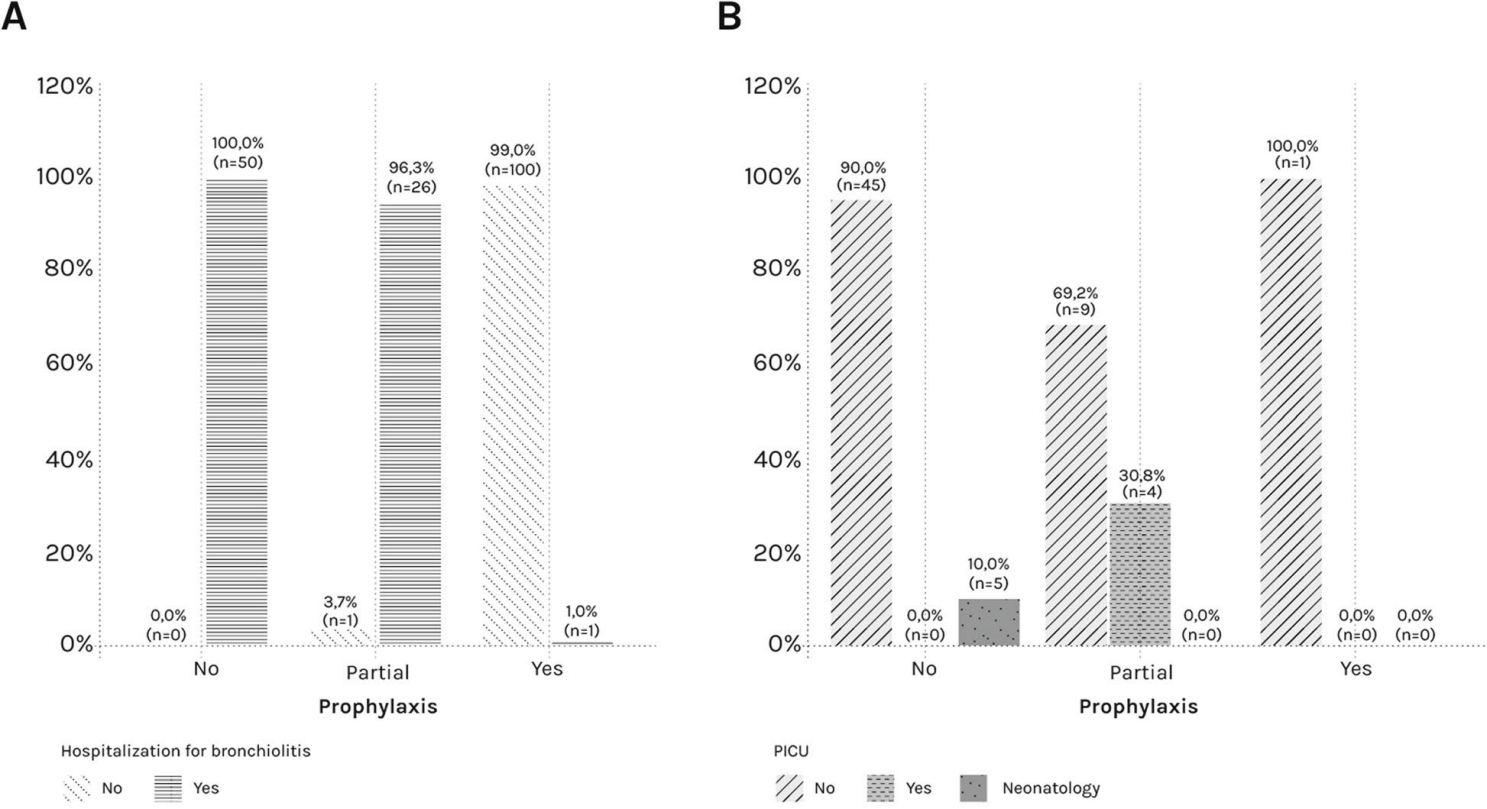
Descriptive statistics of the hospitalization (**A**) and the PICU (**B**). **A** Descriptive statistics of the hospitalization for bronchiolitis in the prophylaxis levels (*n* = 178). **B** Descriptive statistics of the PICU in the prophylaxis levels (*n* = 64). Prophylaxis levels: NO; Partial (≤ 3 doses of palivizumab); Yes (a complete cycle of immunization

This study shows the shielding effect of palivizumab prophylaxis on patients with HS-CHD. and the results are consistent with other previous studies on palivizumab prophylaxis. In the present study of the 128 HS-CHD infants aged from 0 to 24 months who received palivizumab prophylaxis, 102 completed the 5-dose cycle while 26 received incomplete prophylaxis (≤ 3 doses). In this group, 27 patients needed hospitalisation for RSV-infection disease. Noteworthy, nearly all of them (26 out of 27) did not receive a complete cycle of prophylaxis (≤ 3 doses) (Fig. 1A) CHD patients are more likely to have a severe course of RSV-infection disease and thus are at higher risk of increased morbidity and mortality. HS-CHD patients have an altered hemodynamic balance which may be worsened by RSV-infection which causes a ventilation – perfusion mismatch leading to respiratory failure.

In several studies CHD have been found to be an independent risk factor for hospitalisation irrespective of hemodynamic significance [20–23]. Children with HS-CHD with RSV-infection may need hospital admission for prompt supportive care and oxygen therapy. Data reported in literature show high RSV-hospitalization rate in HS-CHD patients even if data present a wide range of variation [22, 24–26]. The length of hospital stay in HS-CHD patients RSV-infected, in our study, was longer compared with CG (14.4 ± 21.7 days vs 6.2 ± 2.3 days; *p*-value˂ 0.0001), and some require admission in PICU, finding similar to that of other studies [27, 28], which may impact health care resource availability and utilisation. During hospitalisation patients may go through comorbidities (bacterial co-infection, or others hospitalisation related complications) which may worsen the course of the disease, protracting hospital stay. The fatality rate in HS-CHD children range from 0 to 3%, due to a more severe course of the RSV infection, particularly in complex CHD, compared to children without CHD; and the mortality rate increases along with the severity of the heart defect [11]. Moreover, RSV infection and consequent hospitalization may delay and complicate corrective heart surgery, increasing CHD-related morbidity in CHD patients [29]. In a study including children who underwent cardiac surgery comparing outcomes for those who acquired RSV infection with those who did not, matched for demographics and physiology of cardiac morphology it was found that RSV infection more than 6 weeks before cardiopulmonary bypass caused significant morbidity [30, 31]. In our study one HS-CHD patient who received complete prophylaxis needed hospitalisation for RSV-bronchiolitis, though the reduced dimension of our sample, this is also consistent with the findings in other studies where the RSV-associated hospitalisations rate for children < 24 months, who received complete prophylaxis is very low [31–35], usually HS-CHD patients with one or more underlying medical conditions (genetic and/or neurological disorders, chronic lung disease). Despite this, data confirm that up to now, palivizumab prophylaxis protects CHD from RSV infection and may be the best strategy for control of morbidity and mortality [36–41].

This retrospective study is subject to several bias and limitations. A part for its retrospective nature, it is a single center study with a relatively small sample, some results depend on documentation accuracy by healthcare providers involved. However, these data have been collected in a well define geographical region which capture a significant number of CHD patients who either received or not RSV prophylaxis, thus can be generalizable to similar center providing care to comparable population.

## Conclusions

Our study provides evidence of the efficacy of palivizumab in protecting HS-CHD patients under the age of 2 years from RSV disease and its life-threatening complications. Our observations strikingly demonstrate the efficacy of complete cycle of palivizumab prophylaxis in preventing hospitalization, showing that even with partial prophylaxis CHD patients go through RSV-infection, need hospitalisation with longer stay than children without underlying medical conditions and may require admission in PICU. Reducing hospitalisation rate, morbidity, and mortality in this category of patients, passive immune prophylaxis with palivizumab may impact healthcare resource availability and utilisation, even if the need of more rigorous studies to address the issue persists, there are studies showing a favourable trend in the cost-utility analyses. Therefore, the current limits of prophylaxis may be questionable and the extension of palivizumab prophylaxis period considered.

## Data Availability

All data generated or analyzed during this study are included in this published article.

## Abbreviations

AC: Complex congenital heart defects with increased pulmonary flow
AIFA: Italian medicine agency
AM: Multiple congenital heart defects with increased pulmonary flow
AS: Simple congenital heart defects with increased pulmonary flow
BC: Complex congenital heart defects with pulmonary hypo-afflux
CC: Complex congenital heart defects
CHD: Congenital heart disease
OR: Odd ratio
PICU: Pediatric intensive care unit
RSS: Respiratory severity score
RSV: Respiratory syncytial virus
SD: Standard deviations
SICP: Italian society for pediatric cardiology and congenital cardiopathies
